# Targeting Periodontitis with Treg-Derived Extracellular Vesicles: Modulation of Macrophages and CD8^+^ T-Cell Responses

**DOI:** 10.3390/ijms27135845

**Published:** 2026-06-29

**Authors:** Carolina Rojas, Luis González-Osuna, Michelle García, Alfredo Sierra-Cristancho, Luis Daniel Sansores-España, Paola Carvajal, Lesley A. Smyth, Karina Pino-Lagos, Rolando Vernal

**Affiliations:** 1 Periodontal Biology Laboratory, Faculty of Dentistry, Universidad de Chile, Santiago 8380544, Chile; luisgodont@gmail.com (L.G.-O.); michelitapao@gmail.com (M.G.); asierra@odontologia.uchile.cl (A.S.-C.); lsansores@odontologia.uchile.cl (L.D.S.-E.); pcarvajal@odontologia.uchile.cl (P.C.); 2 Biomedical Research and Innovation Center, Faculty of Medicine, Universidad de Los Andes, Santiago 7620086, Chile; karina.p.lagos@gmail.com; 3 Department of Growth, Development and Public Health, Faculty of Dentistry, Universidad de los Andes, Santiago 7620086, Chile; 4 Faculty of Dentistry, Universidad Andres Bello, Santiago 8370134, Chile; 5 Department of Conservative Dentistry, Faculty of Dentistry, Universidad de Chile, Santiago 8380544, Chile; 6 School of Medicine and Biomedical Sciences, University of West London, London W5 5RF, UK; lesley.smyth@uwl.ac.uk

**Keywords:** extracellular vesicles, periodontitis, regulatory T cell, Treg, alveolar bone loss, macrophages, CD8^+^ T lymphocytes

## Abstract

Periodontitis is a chronic inflammatory disease characterized by alveolar bone loss driven by dysregulated immune responses. We previously showed that extracellular vesicles derived from retinoic acid-induced regulatory T lymphocytes (RA-Treg EVs) suppress pathogenic CD4^+^ T-lymphocyte responses and reduce alveolar bone loss during periodontitis. Herein, we investigated whether RA-Treg EVs also modulate macrophage and CD8^+^ T-lymphocyte responses during experimental periodontitis. Ligature-induced periodontitis was generated in mice, followed by local administration of RA-Treg EVs. Alveolar bone loss was analyzed by micro-computed tomography, and periodontal tissues and cervical lymph nodes were analyzed by flow cytometry to quantify antigen-presenting cells, macrophages, macrophage subsets, and CD8^+^ T lymphocytes. The direct effects of RA-Treg EVs on macrophage phenotype and CD8^+^ T-cell proliferation and activation were assessed in vitro. RA-Treg EV treatment attenuated alveolar bone loss and preserved trabecular microarchitecture. This effect was associated with reduced macrophage infiltration into periodontal tissues, modulation of macrophage polarization, and restoration of CD8^+^ T-cell abundance in periodontal tissues and draining cervical lymph nodes, without major changes in CD8^+^IFN-γ^+^ or CD8^+^RANKL^+^ cells. In vitro, RA-Treg EVs induced heterogeneous macrophage phenotypes distinct from the classical M1/M2 polarization states while markedly enhancing CD8^+^ T-cell proliferation and activation. These findings indicate that RA-Treg EVs preserve alveolar bone during experimental periodontitis while selectively modulating macrophage and CD8^+^ T-lymphocyte responses.

## 1. Introduction

Periodontitis is a highly prevalent chronic inflammatory disease characterized by the progressive destruction of the tooth-supporting alveolar bone, which, if left untreated, ultimately leads to tooth loss [1]. Periodontitis pathogenesis is primarily driven by a dysbiotic subgingival biofilm attached to the tooth surface [2]. Although bacteria within this biofilm can directly contribute to tissue damage, their pathogenicity largely depends on the dysregulated immune response they trigger in the host’s periodontal tissues [3,4]. Among the key immune cell populations orchestrating this response, macrophages, CD4^+^ T cells, and CD8^+^ T cells play critical roles in shaping the local immune landscape and modulating alveolar bone remodeling [5,6,7].

Within the CD4^+^ T-cell compartment, T helper 17 (Th17) and regulatory T (Treg) cells exert opposing effects on bone homeostasis by controlling the balance between bone resorption and formation [1,5]. Th17 cells drive periodontal inflammation and promote osteoclast differentiation and activation, thereby exacerbating bone loss [1,5]. In contrast, Tregs suppress Th17-mediated inflammation, induce apoptosis of osteoclast precursors, and support bone preservation [1,5]. In this context, CD8^+^ T cells, traditionally recognized for their cytotoxic functions, have emerged as important modulators of osteoimmune interactions, capable of influencing osteoclast activity and bone remodeling in periodontitis [6,8,9]. Similarly, macrophages contribute to this complex interplay through their plasticity, polarizing into pro-inflammatory or anti-inflammatory phenotypes that exert opposing effects on bone homeostasis [7,10,11].

CD8^+^ T lymphocytes are increasingly recognized as functionally heterogeneous cells whose activities extend beyond classical cytotoxicity [12]. In periodontal lesions, CD8^+^ T cells are part of the local adaptive immune infiltrate, and changes in CD8^+^ T-cell abundance and in the CD4^+^/CD8^+^ T-cell ratio have been associated with periodontal inflammation [9,10]. Importantly, non-cytotoxic CD8^+^ T-cell subsets may participate in bone homeostasis through both anti-resorptive and pro-regenerative mechanisms. In experimental periodontitis, CD8^+^Foxp3^+^ regulatory T cells have been shown to reduce alveolar bone destruction and osteoclast formation, at least in part by modulating the local Th17/Treg balance and decreasing pro-osteoclastogenic mediators such as IL-17A and RANKL [8]. In addition, osteoclast-induced regulatory CD8^+^ T cells can suppress osteoclastogenesis and limit bone resorption through mechanisms involving IFN-γ-dependent inhibition of RANKL signaling in osteoclast precursors [13,14]. Beyond their anti-osteoclastogenic activity, CD8^+^ T cells may also promote bone formation. In bone and periodontal regenerative models, CD8^+^ T cells have been identified as a relevant source of Wnt10b, a bone-anabolic Wnt ligand that stimulates osteoblast-lineage cell activity, enhances periodontal ligament cell proliferation and differentiation, and contributes to bone formation [15,16,17,18]. Therefore, CD8^+^ T cells represent a biologically relevant yet comparatively underexplored lymphocyte population in periodontitis, with potential roles in both inflammatory bone resorption and bone repair.

Recent advances in immunotherapy have led to the development of strategies to mitigate alveolar bone loss by modulating immune responses [19,20]. Among these, approaches that enhance regulatory mechanisms have received particular attention for their potential to restore immune homeostasis in periodontal tissues [21,22]. In this context, extracellular vesicles (EVs) derived from Tregs have emerged as a promising cell-free immunomodulatory tool, capable of delivering anti-inflammatory and regulatory signals to target cells [23]. We recently demonstrated that EVs derived from Tregs induced by retinoic acid (RA-Treg EVs) express high levels of the CD73 ectoenzyme, which catalyzes adenosine production and effectively suppresses CD4^+^ T cell-mediated inflammation in periodontitis [3]. In particular, periodontal administration of RA-Treg EVs significantly attenuates Th17-driven inflammation and ameliorates alveolar bone loss [3].

Despite these encouraging results, the mechanisms by which RA-Treg EVs influence other critical immune cell subsets involved in periodontal osteoimmunology, such as macrophages and CD8^+^ T lymphocytes, remain unexplored. Therefore, this study aimed to investigate whether local administration of RA-Treg EVs controls periodontitis-related alveolar bone loss by modulating macrophage and CD8^+^ T-cell responses within periodontal tissues. We hypothesized that RA-Treg EVs modulate the activity of periodontal macrophages and CD8^+^ T lymphocytes, thereby supporting alveolar bone preservation.

## 2. Results

### 2.1. RA-Treg EVs Protect Against Alveolar Bone Loss During Periodontitis

**Figure 1:**
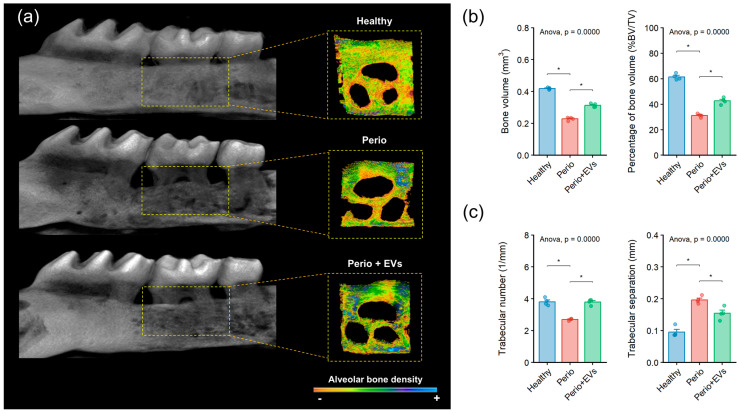
Experimental periodontitis and alveolar bone loss. (**a**) Representative micro-CT images of a non-ligated control mouse (Healthy), a ligature-induced periodontitis mouse (Perio), and a RA-Treg EV-treated periodontitis mouse (Perio + EVs). On the left, the micro-CT-generated volumetric reconstruction shows the volume of interest (VOI) in the maxillary second molar, indicated by a yellow square. On the right, occlusal view of the VOI with digital exclusion of the teeth, showing alveolar bone loss as a color gradient scale. (**b**) Changes in bone quantity are plotted as bone volume (mm^3^) and percentage of bone volume (%BV/TV). (**c**) Changes in bone quality are plotted as trabecular number (1/mm) and trabecular separation (mm). Data are shown as mean ± standard deviation. Statistical analysis was performed using one-way ANOVA followed by Bonferroni’s multiple comparisons test. * *p* < 0.05.

The protective effect of RA-Treg EVs on alveolar bone loss was evaluated in ligature-induced periodontitis in mice using micro-computed tomography (micro-CT) (Figure 1). Healthy non-ligated mice exhibited preserved bone morphology, while periodontitis mice developed significant bone loss surrounding the maxillary molars (Figure 1a). Notably, mice with periodontitis that received local treatment with RA-Treg EVs showed attenuated alveolar bone destruction compared with untreated mice with periodontitis.

Quantitative micro-CT data confirmed that periodontitis led to a significant reduction in bone volume (mm^3^) and percentage of bone volume (%BV/TV), both of which were significantly ameliorated in mice treated with RA-Treg EVs (Figure 1b). Similarly, trabecular analysis revealed a significant decrease in trabecular number (1/mm) and an increase in trabecular separation (mm) in periodontitis-affected mice, consistent with deteriorated bone quality (Figure 1c). These parameters were significantly improved in RA-Treg EV-treated periodontitis mice, indicating that the therapy preserved both the quantity and microarchitectural integrity of alveolar bone. Altogether, these findings demonstrate that local delivery of RA-Treg EVs mitigates alveolar bone loss and preserves bone structure during experimental periodontitis.

### 2.2. RA-Treg EVs Modulate Macrophage Infiltration Without Altering the Overall Abundance of Antigen-Presenting Cells in Periodontal Tissues

**Figure 2:**
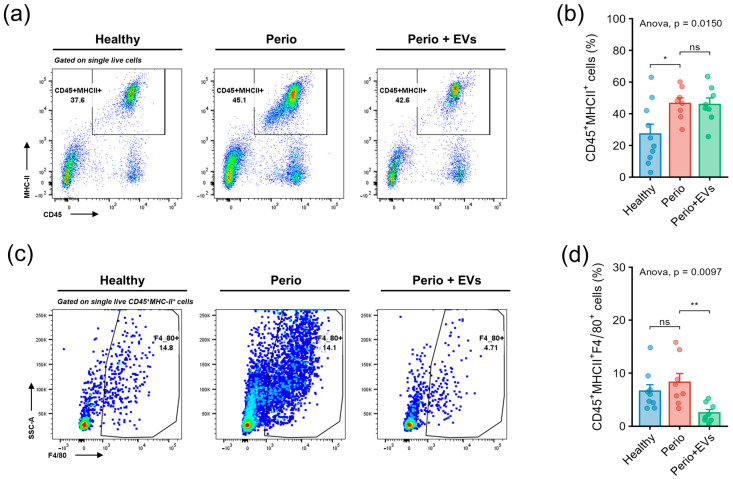
Detection of antigen-presenting cells and macrophages in periodontal tissues. (**a**) Representative flow cytometry dot plots showing the identification of total antigen-presenting cells (APCs), defined as viable CD45^+^MHC class II^+^ cells, infiltrating periodontal tissues from a non-ligated control mouse (Healthy), a ligature-induced periodontitis mouse (Perio), and a RA-Treg EV-treated periodontitis mouse (Perio + EVs). (**b**) Quantification of the frequency of total CD45^+^MHC class II^+^ APCs in periodontal tissues. (**c**) Representative flow cytometry dot plots illustrating the identification of total macrophages, defined as viable CD45^+^MHC class II^+^F4/80^+^ cells, infiltrating periodontal tissues across the experimental groups. (**d**) Quantification of the frequency of total CD45^+^MHC class II^+^F4/80^+^ macrophages in periodontal tissues. Data are shown as mean ± standard deviation. Statistical analysis was performed using one-way ANOVA followed by Bonferroni’s multiple comparisons test. * *p* < 0.05, ** *p* < 0.01, ns: non-significant.

To analyze the impact of RA-Treg EVs on antigen-presenting cells (APCs) within periodontal tissues, flow cytometry was used to quantify total CD45^+^MHC class II^+^ cells (Figure 2). Representative dot plots illustrating the identification of these APCs in non-ligated healthy mice, ligature-induced periodontitis mice, and RA-Treg EV-treated periodontitis mice are shown in Figure 2a. Quantitative analysis revealed a significant increase in the frequency of CD45^+^MHC class II^+^ cells in periodontitis mice compared with healthy controls, indicating enhanced recruitment or activation of antigen-presenting cells during periodontal inflammation (Figure 2b). Notably, local administration of RA-Treg EVs did not significantly alter the proportion of these cells compared with untreated periodontitis mice, suggesting that this therapeutic intervention does not affect the overall abundance of APCs within the periodontal microenvironment.

Given the central role of macrophages in periodontal osteoimmunology, we next assessed the presence of total macrophages defined as CD45^+^MHC class II^+^F4/80^+^ cells (Figure 2). The gating strategy used to identify viable macrophages infiltrating periodontal tissues is shown in Figure S1a. Representative flow cytometry dot plots identifying these macrophages across the experimental groups are shown in Figure 2c. Quantitative analysis showed no significant differences in the total macrophage frequency between healthy and periodontitis mice (Figure 2d). Interestingly, treatment with RA-Treg EVs resulted in a significant reduction in the proportion of CD45^+^MHC class II^+^F4/80^+^ macrophages compared with untreated periodontitis animals, indicating a selective modulatory effect of RA-Treg EVs on macrophage infiltration or persistence within periodontal tissues. Collectively, these findings demonstrate that while experimental periodontitis is associated with an increased number of CD45^+^MHC class II^+^ APCs, RA-Treg EV treatment selectively reduces macrophage infiltration without altering the overall abundance of APCs. This differential modulation highlights the targeted immunoregulatory properties of RA-Treg EVs within the periodontal inflammatory milieu.

### 2.3. RA-Treg EVs Modulate Macrophage Polarization Without Altering the M2/M1 Ratio in Periodontal Tissues

**Figure 3:**
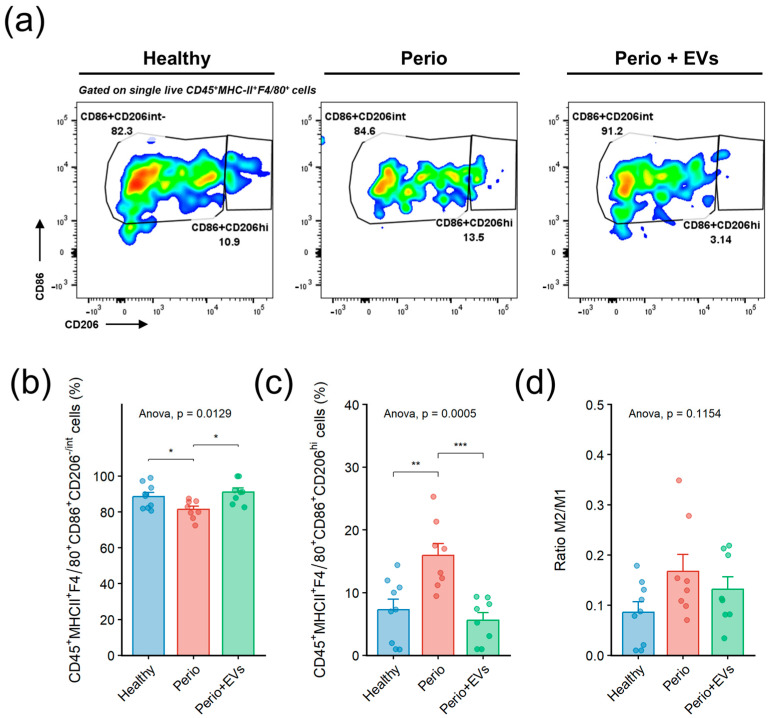
Detection of M1 and M2 macrophage subsets in periodontal tissues. (**a**) Representative flow cytometry dot plots showing the identification of macrophages infiltrating periodontal tissues from a non-ligated control mouse (Healthy), a ligature-induced periodontitis mouse (Perio), and a RA-Treg EV-treated periodontitis mouse (Perio + EVs). Macrophages were defined as viable CD45^+^MHC class II^+^F4/80^+^ cells and further classified, based on CD86 and CD206 expression, into M1-like macrophages (CD86^+^CD206^−/int^) and M2-like macrophages (CD86^+^CD206^hi^). (**b**) Quantification of the frequency of M1-like macrophages (CD45^+^MHC class II^+^F4/80^+^CD86^+^CD206^−/int^) in periodontal tissues. (**c**) Quantification of the frequency of M2-like macrophages (CD45^+^MHC class II^+^F4/80^+^CD86^+^CD206^hi^) in periodontal tissues. (**d**) Quantification of the M2/M1 macrophage ratio across the experimental groups. Data are shown as mean ± standard deviation. Statistical analysis was performed using one-way ANOVA followed by Bonferroni’s multiple comparisons test. * *p* < 0.05, ** *p* < 0.01, *** *p* < 0.001.

To further analyze the immunomodulatory effects of RA-Treg EVs on macrophages during experimental periodontitis, flow cytometry was performed to quantify M1- and M2-like macrophage subsets in periodontal tissues. Macrophages were identified as viable CD45^+^MHC class II^+^F4/80^+^ cells and subsequently classified based on CD86 and CD206 expression into M1-like macrophages (CD86^+^CD206^−/int^) and M2-like macrophages (CD86^+^CD206^hi^). Representative dot plots illustrating the identification of these macrophage subsets across the experimental groups are shown in Figure 3a. Quantitative analysis paradoxically revealed a significant decrease in the frequency of M1-like macrophages in periodontitis mice compared with healthy controls, indicating that experimental periodontitis alters the proportion of this macrophage subset (Figure 3b). Notably, treatment with RA-Treg EVs significantly increased the frequency of M1-like macrophages compared with untreated periodontitis mice, reaching levels similar to those observed in healthy unligated mice. In contrast, the frequency of M2-like macrophages was significantly higher in periodontitis mice than in healthy controls (Figure 3c), suggesting an expansion of this subset in response to periodontal inflammation. Unexpectedly, local administration of RA-Treg EVs reduced the proportion of M2-like macrophages compared with untreated periodontitis mice, bringing their levels closer to those observed in healthy animals.

To assess the overall balance between these macrophage phenotypes, the M2/M1 ratio was calculated (Figure 3d). Despite changes in individual macrophage subsets, the M2/M1 ratio remained comparable across all experimental conditions, indicating that RA-Treg EV treatment does not significantly shift the overall macrophage polarization balance in periodontal tissues. Collectively, these findings demonstrate that experimental periodontitis is associated with an increased proportion of M2-like macrophages, whereas RA-Treg EV treatment modestly increases M1-like macrophages and reduces M2-like macrophages, without significantly affecting the M2/M1 ratio. These results highlight a nuanced, context-dependent immunomodulatory effect of RA-Treg EVs on macrophage polarization within the periodontal inflammatory milieu.

### 2.4. Direct Interaction of RA-Treg EVs with Macrophages Induces Heterogeneous Activation Phenotypes

Given that RA-Treg EV treatment altered the frequency of M1- and M2-like macrophage subsets in vivo without significantly modifying the overall M2/M1 ratio, we next sought to determine whether RA-Treg EVs could directly interact with macrophages and promote activation states not fully captured by the conventional M1/M2 classification or the CD86/CD206 discrimination strategy. To address this question, RAW264.7 macrophages were exposed to DiR-labeled RA-Treg EVs, and the EV-macrophage interaction, as well as its biological consequences, were analyzed by flow cytometry. Following exposure, virtually all DiR-RA-Treg EV-exposed macrophages became DiR-positive, indicating a robust association between RA-Treg EVs and target cells (Figure S2a). Notably, this effect was not detected in DiR-PBS-exposed macrophages. Although these data do not allow discrimination between EV internalization and membrane association, they support a direct interaction between RA-Treg EVs and macrophages.

**Figure 4:**
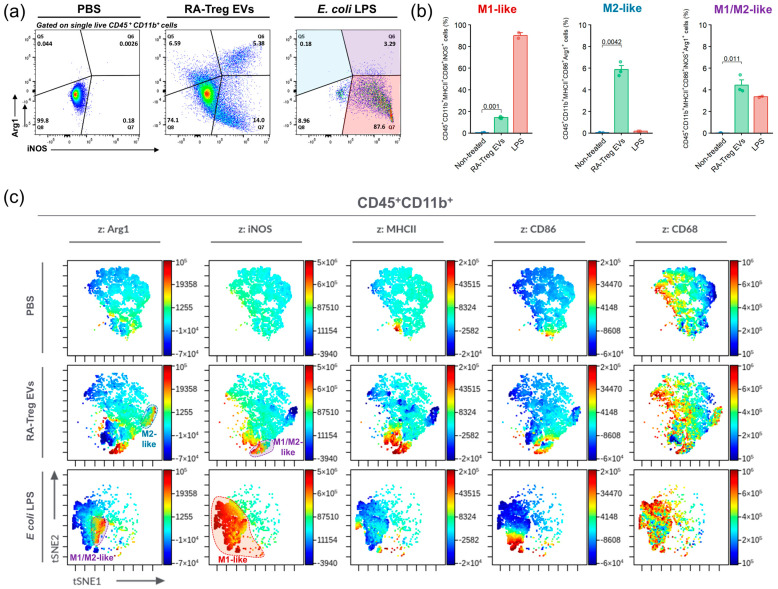
Flow cytometric analysis of RA-Treg EV interaction and macrophage phenotypes in vitro. (**a**) Representative flow cytometry dot plots illustrating the expression of inducible nitric oxide synthase (iNOS) and arginase-1 (Arg1) in RAW264.7 macrophages treated with PBS (Control), *Escherichia coli* LPS, or RA-Treg EVs. Macrophages were classified according to iNOS and Arg1 expression into Arg1^−^iNOS^+^, Arg1^+^iNOS^−^, Arg1^+^iNOS^+^, and Arg1^−^iNOS^−^ populations. (**b**) Quantification of the frequency of M1-like macrophages (CD45^+^CD11b^+^MHC class II^+^CD86^+^iNOS^+^), M2-like macrophages (CD45^+^CD11b^+^MHC class II^+^CD86^+^Arg1^+^), and M1/M2-like macrophages (CD45^+^CD11b^+^MHC class II^+^CD86^+^iNOS^+^Arg1^+^) in the Non-treated (PBS), RA-Treg EV-treated, and *E. coli* LPS-treated conditions. Data are shown as mean ± standard deviation. Statistical analysis was performed using the Wilcoxon rank-sum test. (**c**) Unsupervised t-distributed stochastic neighbor embedding (t-SNE) analysis of RAW264.7 macrophages based on the combined expression of Arg1, iNOS, MHC class II, CD86, and CD68. Individual panels depict the distribution of macrophages across treatment conditions (PBS, RA-Treg EVs, or *E. coli* LPS).

To investigate whether this interaction translated into functional phenotypic changes, macrophage responses to RA-Treg EVs were characterized using a broader panel of polarization-related markers, including inducible nitric oxide synthase (iNOS), arginase-1 (Arg1), CD86, MHC-II, and CD68. Additionally, macrophages stimulated with *Escherichia coli* lipopolysaccharide (LPS), a well-known pro-inflammatory stimulus, were analyzed as a reference (Figure 4). The gating strategy used to identify polarization marker expression in viable macrophages is shown in Figure S2b. As expected, *E. coli* LPS exposure predominantly induced an iNOS^+^Arg1^−^ population, consistent with a classical M1-like inflammatory phenotype, together with a smaller population of iNOS^+^Arg1^+^ cells exhibiting a hybrid M1/M2-like phenotype. In contrast, RA-Treg EV-treated macrophages displayed a markedly heterogeneous response characterized by the simultaneous emergence of Arg1^−^iNOS^+^, Arg1^+^iNOS^−^, and Arg1^+^iNOS^+^ populations (Figure 4a,b). These findings suggest that RA-Treg EVs do not simply promote a shift toward either canonical M1 or M2 polarization, but rather induce a spectrum of macrophage phenotypes with features of both programs.

Moreover, to resolve this heterogeneity, unsupervised t-distributed stochastic neighbor embedding (t-SNE) analysis was performed using the combined expression of iNOS, Arg1, CD86, MHC-II, and CD68 (Figure 4c). Remarkably, macrophages exposed to RA-Treg EVs occupied phenotypic regions distinct from those induced by *E. coli* LPS, while partially overlapping with PBS-treated macrophages. In particular, iNOS-expressing macrophages generated following RA-Treg EV exposure clustered separately from the canonical LPS-induced inflammatory population and exhibited distinct MHC-II, CD86, and CD68 expression profiles. Notably, marked MHC-II expression colocalized with the Arg1-enriched population. In contrast, *E. coli* LPS induced higher levels of the co-stimulatory molecule CD86 and the phagocytic/activation marker CD68, which colocalized with the iNOS^+^ M1-like population. Moreover, Arg1^+^iNOS^+^ cells induced by RA-Treg EVs localized within an intermediate phenotypic space between classical M1- and M2-like populations, whereas *E. coli* LPS-induced Arg1^+^iNOS^+^ cells localized within the iNOS^+^ M1-like population, supporting the notion that RA-Treg EVs induce the emergence of a unique hybrid macrophage phenotype. Collectively, these findings provide evidence that RA-Treg EVs directly interact with macrophages and promote unique activation programs that extend beyond the classical M1/M2 paradigm, potentially explaining the subtle and context-dependent effects observed in periodontal tissues in vivo.

### 2.5. RA-Treg EVs Restore Non-Cytotoxic, Non-Osteoclastogenic CD8^+^ T Lymphocyte Infiltration in Periodontal Tissues

**Figure 5:**
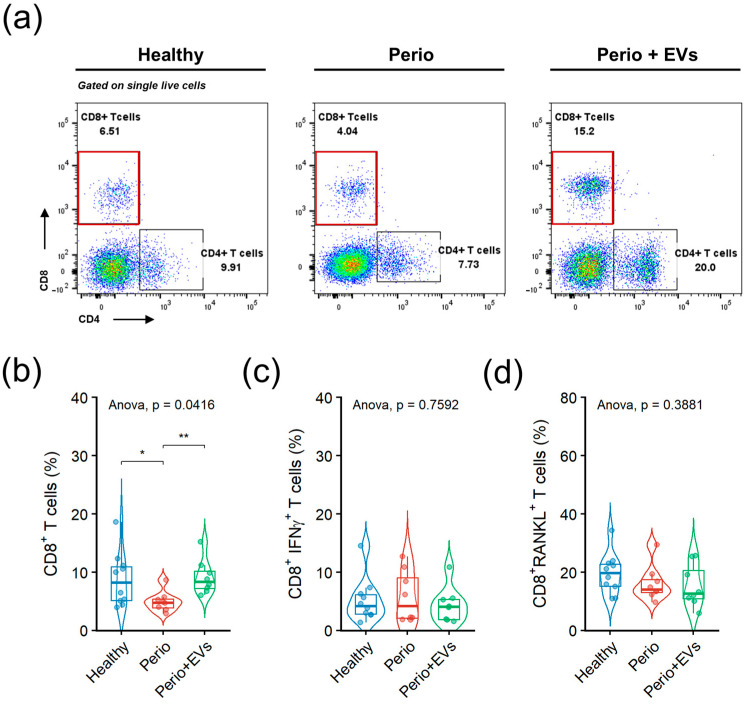
Detection of CD8^+^ T lymphocytes in periodontal tissues. (**a**) Representative flow cytometry dot plots showing the identification of total CD8^+^ T lymphocytes infiltrating periodontal tissues from a non-ligated control mouse (Healthy), a ligature-induced periodontitis mouse (Perio), and a RA-Treg EV-treated periodontitis mouse (Perio + EVs). Red gates highlight the CD8^+^ T lymphocyte population selected for analysis. (**b**) Quantification of the frequency of total CD8^+^ T lymphocytes in periodontal tissues. (**c**) Quantification of the frequency of CD8^+^ T lymphocytes expressing IFN-γ. (**d**) Quantification of the frequency of CD8^+^ T lymphocytes expressing RANKL. Data are presented as violin plots showing the median, with boxes representing the interquartile range and whiskers indicating the 10th and 90th percentiles. Statistical analysis was performed using one-way ANOVA with Welch’s correction, followed by Bonferroni’s multiple comparisons test. * *p* < 0.05 and ** *p* < 0.01.

To analyze whether RA-Treg EVs influence CD8^+^ T-cell responses during periodontitis, the presence of infiltrating CD8^+^ T lymphocytes in periodontal tissues was analyzed by flow cytometry (Figure 5). The gating strategy used to identify viable CD45^+^CD8^+^ T lymphocytes infiltrating periodontal tissues is shown in Figure S1b.

Representative dot plots illustrating the identification of total CD8^+^ T lymphocytes in each experimental condition are shown in Figure 5a. Quantitative analysis revealed that healthy non-ligated mice exhibited the highest frequency of periodontal CD8^+^ T lymphocytes (Figure 5b). In contrast, mice with ligature-induced periodontitis displayed a significant reduction in the proportion of CD8^+^ T lymphocytes. Notably, local administration of RA-Treg EVs significantly increased the frequency of CD8^+^ T lymphocytes compared with untreated periodontitis mice, partially restoring their levels toward those observed in healthy animals.

To further assess the functional phenotype of infiltrating CD8^+^ T lymphocytes, intracellular expression of the pro-inflammatory cytokine interferon-γ (IFN-γ) and the pro-osteolytic mediator termed receptor activator of nuclear factor κB ligand (RANKL) was evaluated. The proportion of CD8^+^ T lymphocytes expressing IFN-γ did not differ significantly among healthy, periodontitis, and RA-Treg EV-treated animals (Figure 5c), indicating that neither the disease condition nor the therapeutic intervention altered their pro-inflammatory cytokine profile. Similarly, the frequency of CD8^+^ T lymphocytes expressing RANKL was comparable across all experimental conditions (Figure 5d), suggesting that the osteoclastogenic potential of these cells remained unchanged. Collectively, these results demonstrate that experimental periodontitis is associated with reduced infiltration of CD8^+^ T lymphocytes into periodontal tissues and that treatment with RA-Treg EVs restores their infiltration. However, this modulation occurs without significant changes in cytotoxic or osteoclastogenic CD8^+^ T cell subsets, as evidenced by unchanged expression of IFN-γ and RANKL.

### 2.6. RA-Treg EVs Modulate CD8^+^ T Lymphocyte Responses in Cervical Lymph Nodes

**Figure 6:**
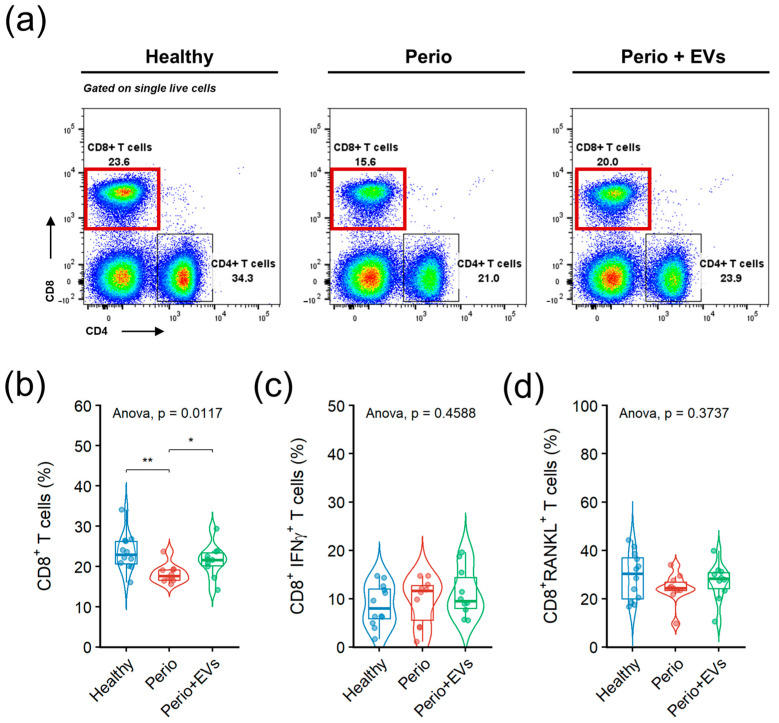
Detection of CD8^+^ T lymphocytes in cervical lymph nodes draining periodontal tissues. (**a**) Representative flow cytometry dot plots showing the identification of total CD8^+^ T lymphocytes in cervical lymph nodes from a non-ligated control mouse (Healthy), a ligature-induced periodontitis mouse (Perio), and a RA-Treg EV-treated periodontitis mouse (Perio + EVs). Red gates highlight the CD8^+^ T lymphocyte population selected for analysis. (**b**) Quantification of the frequency of total CD8^+^ T lymphocytes in cervical lymph nodes. (**c**) Quantification of the frequency of CD8^+^ T lymphocytes expressing IFN-γ. (**d**) Quantification of the frequency of CD8^+^ T lymphocytes expressing RANKL. Data are presented as violin plots showing the median, with boxes representing the interquartile range and whiskers indicating the 10th and 90th percentiles. Statistical analysis was performed using one-way ANOVA with Welch’s correction, followed by Bonferroni’s multiple comparisons test. * *p* < 0.05 and ** *p* < 0.01.

To further characterize CD8^+^ T lymphocyte responses associated with experimental periodontitis and RA-Treg EV treatment, an additional phenotypic analysis was performed in cervical lymph nodes draining the periodontal tissues, owing to the higher cellular yield from these lymphoid organs (Figure 6). The gating strategy used to identify viable CD45^+^CD8^+^ T lymphocytes is shown in Figure S1c.

Representative flow cytometry dot plots illustrating the identification of total CD8^+^ T lymphocytes in each experimental condition are shown in Figure 6a. Consistent with the findings observed in periodontal tissues, quantitative analysis revealed a significant reduction in the frequency of CD8^+^ T lymphocytes in periodontitis mice compared with healthy controls (Figure 6b). Notably, treatment with RA-Treg EVs significantly increased the proportion of CD8^+^ T lymphocytes relative to untreated periodontitis mice, restoring their levels to those observed in healthy animals. In addition, consistent with the findings in periodontal tissues, the proportions of CD8^+^IFN-γ^+^ T cells (Figure 6c) and CD8^+^RANKL^+^ T cells (Figure 6d) did not differ among the Healthy, Perio, and Perio + EVs groups in cervical lymph nodes. Collectively, these findings demonstrate that RA-Treg EVs restore both local and regional CD8^+^ T lymphocyte levels during periodontitis, and that cervical lymph nodes recapitulate the changes observed in periodontal tissues in terms of CD8^+^ T lymphocyte abundance.

### 2.7. RA-Treg EVs Enhance CD8^+^ T-Cell Proliferation and Activation In Vitro

**Figure 7:**
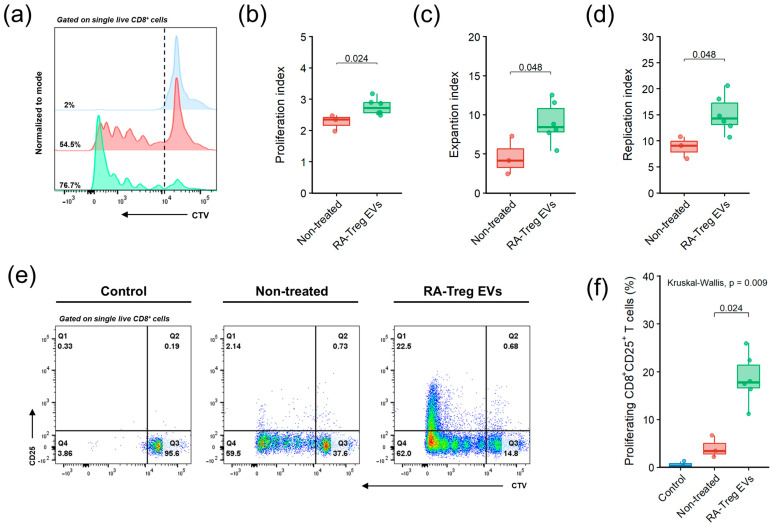
CD8^+^ T-lymphocyte proliferation and activation in vitro. (**a**) Representative flow cytometry histograms showing the proliferation of CD8^+^ T lymphocytes assessed by CellTrace™ Violet (CTV) dilution under three experimental conditions: non-activated T cells (Control, blue), anti-CD3ε-activated T cells exposed to vehicle PBS without RA-Treg EVs (Non-treated, red), and anti-CD3ε-activated T cells treated with RA-Treg EVs (RA-Treg EVs, green). (**b**) Quantification of the proliferation index of CD8^+^ T lymphocytes in the Non-treated and RA-Treg EV-treated conditions. (**c**) Quantification of the expansion index of CD8^+^ T lymphocytes in the Non-treated and RA-Treg EV-treated conditions. (**d**) Quantification of the replication index of CD8^+^ T lymphocytes in the Non-treated and RA-Treg EV-treated conditions. (**e**) Representative flow cytometry dot plots illustrating the percentage of activated CD8^+^ T lymphocytes based on CD25 expression across the experimental conditions (Control, Non-treated, and RA-Treg EVs). (**f**) Quantification of the frequency of CD8^+^CD25^+^ T lymphocytes in the indicated experimental groups. Data are presented as boxplots showing the median, with boxes representing the interquartile range and whiskers indicating the 10th and 90th percentiles. Statistical comparisons in panels (**b**–**d**) were performed using the Mann–Whitney U test. Statistical analysis for panel (**f**) was performed using the Kruskal–Wallis signed-rank test, followed by the Wilcoxon matched-pairs test.

To investigate the direct effects of RA-Treg EVs on CD8^+^ T lymphocytes, in vitro proliferation and activation assays were performed and analyzed by flow cytometry. CD8^+^ T-cell proliferation was assessed by CellTrace™ Violet (CTV; Thermo Fisher Scientific, Rockford, IL, USA) dilution following polyclonal activation with soluble anti-CD3ε. The gating strategy used to identify viable CD8^+^ T lymphocytes is shown in Figure S2c. Representative flow cytometry histograms illustrating CD8^+^ T-cell proliferation under the different experimental conditions are shown in Figure 7a. The experimental conditions included non-activated control cells (Control, blue), anti-CD3ε-activated cells exposed to vehicle PBS without EVs (Non-treated, red), and anti-CD3ε-activated cells treated with RA-Treg EVs (RA-Treg EVs, green). Quantitative analysis of proliferation revealed that RA-Treg EV treatment significantly enhanced CD8^+^ T-cell proliferative responses compared with untreated activated cells. Specifically, the proliferation index (Figure 7b), expansion index (Figure 7c), and replication index (Figure 7d) were significantly higher in RA-Treg EV-treated CD8^+^ T cells than in untreated cells.

To further assess CD8^+^ T-cell activation, the expression of the activation marker CD25 was evaluated by flow cytometry. Representative dot plots depicting CD25 expression on CD8^+^ T lymphocytes across the three experimental conditions are shown in Figure 7e. Quantification of CD8^+^ T-cell activation demonstrated a significant increase in the frequency of CD8^+^CD25^+^ T lymphocytes in the RA-Treg EV-treated condition compared with the non-treated group (Figure 7f). Collectively, these findings demonstrate that RA-Treg-derived EVs directly promote CD8^+^ T-cell proliferation and activation in vitro, highlighting their capacity to finely modulate adaptive immune responses and suggesting a potential mechanism contributing to the restoration of CD8^+^ T-lymphocyte infiltration observed in vivo.

## 3. Discussion

In the present study, we demonstrate that local administration of RA-Treg EVs attenuates ligature-induced alveolar bone loss and modulates key immune cell populations associated with periodontal inflammation. Specifically, RA-Treg EV treatment reduced macrophage infiltration in periodontal tissues and promoted the in vitro induction of heterogeneous macrophage populations, which exhibited unique M1-, M2-, and hybrid M1/M2-like phenotypic features. In parallel, RA-Treg EVs restored the frequency of CD8^+^ T lymphocytes in both periodontal tissues and draining cervical lymph nodes, and directly promoted CD8^+^ T-cell proliferation and activation in vitro. Notably, these effects in periodontal tissues occurred without major changes in the overall abundance of M2/M1 macrophages or in the expression of IFN-γ and RANKL by CD8^+^ T cells. Taken together, these findings indicate that the protective effect of RA-Treg EVs in experimental periodontitis is associated with a selective, nuanced immunomodulatory action rather than broad suppression of immune cell responses, thereby expanding our understanding of how Treg-derived EVs may preserve alveolar bone by regulating macrophage and CD8^+^ T-lymphocyte dynamics.

The bone-protective effect observed in the present study is fully consistent with our previous report, which showed that local administration of RA-Treg EVs ameliorated periodontitis-related alveolar bone loss by reducing both the area of bone resorption and the CEJ-alveolar bone crest distance in molars with periodontal lesions [3]. The present findings extend that evidence by demonstrating, using micro-CT, that the protective effect of RA-Treg EVs is reflected in improved three-dimensional bone quantity and microarchitectural integrity, as indicated by preservation of bone volume, percentage of bone volume, trabecular number, and trabecular separation. Taken together, these complementary datasets strongly support the concept that the bone-preserving activity of RA-Treg EVs is robust across distinct analytical platforms and is not a method-dependent observation but rather a genuine attenuation of inflammatory alveolar bone destruction in experimental periodontitis [3]. This interpretation is biologically coherent with our previous demonstration that RA-Treg EVs suppressed activated, IL-17A^+^, and RANKL^+^ CD4^+^ T-cell responses while reducing osteoclastogenesis, thereby identifying a plausible immunological basis for the anti-resorptive effects confirmed here by micro-CT [3].

Our macrophage-related findings suggest that RA-Treg EVs do not exert a broad suppressive effect on all periodontal APCs, but rather a more selective immunomodulatory action on the macrophage compartment. Indeed, although ligature-induced periodontitis increased the overall frequency of CD45^+^MHC class II^+^ APCs, RA-Treg EV treatment did not significantly modify this population. In contrast, RA-Treg EVs selectively reduced total CD45^+^MHC class II^+^F4/80^+^ macrophages, supporting the idea that these EVs modulate macrophage infiltration or persistence within inflamed periodontal tissues rather than indiscriminately depleting APCs. This observation is consistent with the central osteoimmunological role of macrophages in periodontitis, where they not only act as innate effector and APCs but also shape inflammatory tone, osteoclastogenesis, and downstream adaptive immune responses [10,11,24].

Particularly intriguing were the polarization data, since periodontitis in our model was associated with a higher proportion of M2-like macrophages and a lower proportion of M1-like macrophages, whereas RA-Treg EV treatment partly reversed this pattern without significantly altering the overall M2/M1 ratio. Although this result may appear counterintuitive under the conventional view that M2 macrophages are predominantly protective, increasing evidence indicates that macrophage polarization in periodontal lesions is more complex than a strictly beneficial M2 versus deleterious M1 dichotomy, and that certain M2-associated states may, under specific inflammatory conditions, contribute to tissue remodeling programs or even exhibit enhanced osteoclastogenic potential relative to M1 macrophages [11,24,25]. Accordingly, the reduction in M2-like macrophages observed after RA-Treg EV treatment should not necessarily be interpreted as a loss of a reparative response, but rather as a normalization of a dysregulated macrophage landscape. This interpretation should nevertheless be made with caution, because there is still no universally accepted marker set for defining macrophage subsets in vivo in periodontitis, and the CD86/CD206 strategy used here, while informative and widely employed, captures only a simplified approximation of a highly plastic and continuous spectrum of activation states [10,11,24,26].

Our in vitro experiments further support the notion that the macrophage response to RA-Treg EVs extends beyond the classical M1/M2 polarization paradigm. Exposure of RAW264.7 macrophages to RA-Treg EVs induced heterogeneous populations characterized by distinct combinations of Arg1, iNOS, MHC-II, CD86, and CD68 expression, markedly different from the canonical inflammatory phenotype elicited by *E. coli* LPS. In particular, t-SNE analysis identified Arg1^+^iNOS^+^ hybrid macrophage populations occupying phenotypic regions distinct from those induced by LPS and that preferentially colocalized with MHC class II^high^ cells. In contrast, LPS-induced iNOS^+^ macrophages were primarily associated with CD68^high^ and CD86^high^ expression profiles. Beyond its conventional role as an M2 marker, Arg1 has been implicated in regulatory and tissue-reparative programs by modulating L-arginine metabolism and suppressing excessive inflammation [27], suggesting that the Arg1^+^iNOS^+^ populations induced by RA-Treg EVs may retain regulatory functions while preserving selected inflammatory or antigen-presenting characteristics. Likewise, the distinct expression patterns of CD68, a lysosomal glycoprotein associated with phagocytic activity and inflammatory macrophage responses [28,29], and CD86, a canonical co-stimulatory molecule involved in antigen presentation and commonly associated with M1-like polarization [30], further indicate that iNOS expression alone is insufficient to define a conventional inflammatory macrophage phenotype. Collectively, these findings support the concept that macrophage activation exists along a multidimensional continuum rather than as discrete M1 and M2 states and suggest that RA-Treg EVs promote macrophage reprogramming toward unique hybrid phenotypes. Such phenotypic plasticity may explain why RA-Treg EV treatment modified macrophage subsets in vivo without substantially altering the overall M2/M1 ratio and could contribute to the anti-inflammatory and bone-protective effects observed during experimental periodontitis.

The CD8^+^ T-cell findings in the present study suggest that RA-Treg EVs modulate this compartment in a selective rather than broadly activating manner. In both periodontal tissues and draining cervical lymph nodes, RA-Treg EV treatment restored the frequency of total CD8^+^ T lymphocytes. Nevertheless, this effect was not accompanied by detectable changes in the proportions of CD8^+^IFN-γ^+^ or CD8^+^RANKL^+^ cells, indicating that the increase in total CD8^+^ T cells is unlikely to reflect a simple expansion of classical pro-inflammatory or pro-osteoclastogenic effector subsets [6,9]. This pattern is consistent with the recognized functional heterogeneity of CD8^+^ T cells in periodontitis, in which these lymphocytes may exert not only cytotoxic and inflammatory activities but also regulatory and potentially tissue-protective functions [8,9]. Accordingly, one plausible interpretation is that RA-Treg EVs may favor the accumulation, survival, or activation of alternative CD8^+^ T-cell subsets not assessed in the present study. In this regard, an attractive hypothesis for future investigation is that RA-Treg EVs could promote osteoanabolic CD8^+^ T cells capable of producing Wnt10b. This Wnt ligand has been shown in other skeletal settings to stimulate canonical Wnt/β-catenin signaling, enhance Runx2-dependent osteoblastogenesis, and support bone formation [15,16,31]. Notably, CD8^+^ T-cell-derived Wnt10b has also been implicated in the regulation of human periodontal ligament cell proliferation and differentiation [17].

The in vitro experiments provide important functional support for the interpretation that RA-Treg EVs do not merely preserve existing CD8^+^ T-cell numbers in vivo, but can directly modulate CD8^+^ T-cell behavior. Specifically, RA-Treg EVs increased the proliferation, expansion, and replication indices of anti-CD3ε-activated CD8^+^ T cells, while also enhancing the frequency of CD8^+^CD25^+^ cells, indicating that these EVs enhance both proliferative responsiveness and activation under polyclonal stimulation. This observation is noteworthy because it contrasts with the predominantly suppressive effects classically attributed to Treg-derived EVs on conventional CD4^+^ T cells, thereby reinforcing the concept that the biological effects of Treg-derived EVs are highly context-, cargo-, and target cell-dependent [3,32,33,34]. In this framework, the present in vitro data are fully compatible with our in vivo findings, since they provide a plausible mechanistic basis for the restoration of total CD8^+^ T-cell frequencies in periodontal tissues and cervical lymph nodes, while also supporting the possibility that RA-Treg EVs favor the expansion of non-classical CD8^+^ T-cell subsets distinct from IFN-γ^+^ and RANKL^+^ effectors. Rather than inducing a globally pro-inflammatory CD8^+^ T-cell response, RA-Treg EVs may therefore promote a qualitatively different activation state, potentially linked to regulatory, reparative, or osteoanabolic functions that should be defined in future studies.

A plausible mechanism underlying these effects is that RA-Treg EVs act as local paracrine immunomodulatory mediators within the periodontal microenvironment. EVs can regulate recipient cells through complementary mechanisms, including interactions between surface molecules and receptors or enzymatic substrates on target cells, direct membrane-associated signaling, and the delivery of intravesicular cargo, such as proteins, lipids, enzymes, and regulatory RNAs, upon EV uptake [23,32,33,34,35,36]. In the case of RA-Treg EVs, our previous work showed that these vesicles are enriched in enzymatically active CD73, which hydrolyzes extracellular 5′-AMP to adenosine, thereby contributing to the suppression of effector CD4^+^ T-cell responses [3]. Since adenosine can limit T-cell activation and inflammatory cytokine production, and may also restrain osteoclast differentiation and activity, CD73/adenosine-dependent signaling represents a biologically plausible pathway by which locally administered RA-Treg EVs attenuate periodontal inflammation and alveolar bone resorption [3,23,32]. In the present study, RA-Treg EV treatment was also associated with reduced macrophage infiltration and restoration of CD8^+^ T-cell abundance in periodontal tissues and draining cervical lymph nodes. These findings suggest that RA-Treg EVs may reshape the periodontal osteoimmune microenvironment by limiting macrophage-driven inflammatory amplification while supporting the persistence or expansion of CD8^+^ T-cell populations that do not exhibit increased IFN-γ or RANKL expression. Nevertheless, the direct contribution of CD8^+^ T cells to alveolar bone preservation remains to be demonstrated. Future studies should define the molecular cargo of RA-Treg EVs, their target-cell uptake or receptor-mediated interactions, and the functional phenotype of the CD8^+^ T-cell subsets promoted by this treatment.

Taken together, the present findings support a model in which locally administered RA-Treg EVs attenuate experimental periodontitis not through indiscriminate immunosuppression, but through selective remodeling of the periodontal osteoimmune microenvironment. In this model, RA-Treg EVs preserve alveolar bone and trabecular microarchitecture while concomitantly reducing total macrophage infiltration and restoring CD8^+^ T-cell abundance. When considered together with the in vitro evidence showing direct enhancement of Arg1^+^ macrophages and CD8^+^ T-cell proliferation and activation, these data suggest that RA-Treg EVs may promote a qualitatively distinct immune state compatible with restrained osteoclastogenic inflammation and potentially improved reparative or osteoanabolic signaling within periodontal tissues [8,10]. However, important limitations should be acknowledged. This study was not designed to define the molecular cargo or receptor-ligand interactions responsible for the effects of RA-Treg EVs on macrophages and CD8^+^ T lymphocytes. In addition, in vivo macrophage polarization was assessed using the CD86/CD206 framework, which, although widely used, does not capture the full phenotypic and functional diversity of macrophage states [11,24]. Finally, the CD8^+^ T-cell analysis was restricted to total cells and two effector-associated markers, IFN-γ and RANKL, so the possible involvement of regulatory or osteoanabolic CD8^+^ T-cell subsets remains unresolved. These issues should be addressed in future studies using deeper phenotypic profiling and targeted functional approaches.

## 4. Materials and Methods

### 4.1. Animals

Male and female ~8-week-old mice were used in this study. To obtain RA-Treg EVs, C57BL/6 Foxp3^GFP+^ transgenic mice (The Jackson Laboratory, Bar Harbor, ME, USA), which express Green Fluorescent Protein (GFP) under the control of the Foxp3 promoter, were used. To induce periodontitis and to obtain responder T lymphocytes, C57BL/6 wild-type mice were used. Animals were maintained under specific pathogen-free conditions at the Institutional Animal Facilities of the Faculty of Medicine, Universidad de Los Andes, or the Faculty of Dentistry, Universidad de Chile. Environmental conditions were strictly controlled, with a 12 h light/dark cycle, lights on at 07:00, an ambient temperature of 24 ± 0.5 °C, a relative humidity of 40–70%, and a ventilation rate of 15 air changes per hour. Mice were housed in gender-specific cages, with ad libitum access to sterile food and water. All female mice used were confirmed to be in non-estrus stages during the experimental procedures. All animal procedures were reviewed and approved by the Institutional Animal Care and Use Committees (Protocols CEC2021017, 18173-ODO-UCH, and 22578-ODO-UCH). The study was conducted in full accordance with national and international ethical standards, including the ARRIVE guidelines, the Council of the American Psychological Society, and the American Veterinary Medical Association (AVMA) recommendations for the care and use of laboratory animals.

### 4.2. RA-Treg EVs

RA-Treg EVs were obtained and characterized as previously described [3]. In brief, CD4^+^ T cells were obtained from the spleens of C57BL/6 Foxp3^GFP+^ mice and cultured under polarizing conditions to promote the generation of RA-induced Tregs. For this, cells were cultured in RPMI-1640 10% exosome-depleted fetal bovine serum (FBS; Thermo Fisher Scientific), supplemented with 10 ng/mL transforming growth factor-β1 (TGF-β1; Thermo Fisher Scientific), 100 IU/mL interleukin-2 IL-2 (IL-2; Thermo Fisher Scientific), and 10 nM retinoic acid (RA; Sigma-Aldrich, Saint Louis, MO, USA), in 24-well plates coated with 10 µg/mL anti-CD3ε (BioXCell, Lebanon, NH, USA) and 1 µg/mL anti-CD28 (Biolegend, San Diego, CA, USA) for 5 days. After induction, culture supernatants were collected, centrifuged at 300× *g* for 5 min, 2000× *g* for 20 min, and twice at 10,000× *g* for 30 min to remove cells and debris, and then ultracentrifuged at 100,000× *g* for 90 min at 4 °C to isolate the RA-Treg EVs. The final RA-Treg EV pellet was washed in filtered PBS and stored at −80 °C until use. As previously reported by our group, RA-Treg EV preparations obtained using this protocol were characterized by nanoparticle tracking analysis (NTA, NanoSight NS300, Malvern Panalytical, Malvern, UK), transmission electron microscopy (Talos F200C G2, ThermoFisher Scientific, Waltham, MA, USA), imaging flow cytometry (Amnis ImageStreamX Mk II, Cytek Biosciences, Fremont, CA, USA), bead-assisted flow cytometry, and Western blot analysis [3]. Nanoparticle tracking analysis showed that most particles ranged between 100 and 200 nm, with a mean size of 141 ± 71 nm, whereas transmission electron microscopy confirmed the presence of vesicular structures compatible with EV morphology. In addition, RA-Treg EVs expressed the canonical EV markers CD9 and CD81, and complementary analyses confirmed the presence of the immunoregulatory ectoenzyme CD73 on these EVs.

### 4.3. Periodontitis Induction and Treatment with RA-Treg EVs

C57BL/6 wild-type mice were randomly assigned to the experimental and control groups. Group 1: Non-ligated healthy mice; Group 2: Untreated ligature-induced periodontitis mice; and Group 3: RA-Treg-EV-treated periodontitis mice. Experimental periodontitis was induced by placement of sterile 5-0 silk ligatures around the maxillary second molars, following a previously established protocol [37]. Ligatures were carefully tied under ketamine/xylazine anesthesia, avoiding trauma to the periodontal tissues, and maintained in place for 10 days to promote localized periodontal inflammation and alveolar bone loss. Non-ligated mice were considered healthy controls. Treatment consisted of injections of 5 μL PBS containing 1 × 10^8^ RA-Treg EVs, administered on days 3 and 6 post-ligature. The RA-Treg EV dose and administration schedule were selected based on our previous in vivo data demonstrating immunomodulatory efficacy and attenuation of alveolar bone loss in the same ligature-induced murine model of periodontitis [3]. Injections were performed into the palatal mucosa adjacent to the second maxillary molars using a calibrated microsyringe (Hamilton Company, Reno, NV, USA) under stereoscopic visualization. Untreated periodontitis mice received equivalent PBS injections alone, without EVs. RA-Treg EV dose and frequency were selected based on our previous data demonstrating in vivo immunomodulatory efficacy [3]. At the end of the treatment period, animals were euthanized by a single intraperitoneal overdose of ketamine/xylazine, and samples of palatal maxillae, periodontal tissues, and cervical lymph nodes were collected for subsequent analyses.

### 4.4. Sample Processing

After euthanasia, the maxillae were mechanically dissected free of soft tissues and subsequently analyzed by micro-CT. Cells from periodontal tissues and cervical lymph nodes were collected for flow cytometry characterization. Palatal periodontal tissues were carefully dissected from the maxillae and immediately digested in RPMI-1640 medium containing 3.2 mg/mL collagenase IV (Gibco Invitrogen Corp., Grand Island, NY, USA) and 0.15 mg/mL DNase I (Merck, Rahway, NJ, USA) for 1 h at 37 °C under gentle agitation, with EDTA added during the last 5 min of incubation. The resulting cell suspensions were filtered through a 70 μm nylon cell strainer. Cervical lymph nodes were directly disaggregated by passing them through a 70 μm nylon cell strainer. The cells obtained were washed twice with PBS containing 2% FBS (Thermo Fisher Scientific), quantified with an automated cell counter (Luna II, Logos Biosystems, Annandale, VA, USA), and immediately subjected to flow cytometry analysis.

### 4.5. Alveolar Bone Loss

Alveolar bone loss was quantified using micro-CT as previously described [38,39]. Maxillae were scanned using a high-resolution micro-CT system (SkyScan 1272, Bruker, Kontich, Belgium) at an isotropic voxel size of 10 μm, with settings of 50 kV and 200 μA. Three-dimensional reconstructions were generated using NRecon software v.1.6.9 (Bruker) and reoriented with DataViewer software v.1.4.4 (Bruker) to standardize the specimen position prior to analysis. A standardized volume of interest (VOI) was demarcated from a horizontal line crossing the cement-enamel junction (CEJ) of the second maxillary molar, a parallel line localized 0.8 mm apical to the CEJ line, in accordance with the apex of the distal root of the third molar, a vertical line tangential to the distal surface of the first molar, and a vertical line tangential to the mesial surface of the third molar. Alveolar bone changes within the VOI were calculated using CTAn software v.2.2.10 (Bruker), and bone loss was expressed as bone volume (mm^3^), percentage of bone volume (%BV/TV), trabecular number (1/mm), and trabecular separation (mm). In addition, a region of interest (ROI) corresponding to the alveolar bone, excluding the teeth, was created in the transverse plane of each set of images. All image acquisition and analyses were performed in a blinded manner by a single examiner.

### 4.6. Macrophage and CD8^+^ T-Cell Detection and Quantification

To evaluate macrophage and CD8^+^ T lymphocyte infiltration within periodontal tissues or cervical lymph nodes, flow cytometry analysis was performed as previously described [39]. Separately, 5 × 10^5^ cells (periodontal tissues) or 1 × 10^6^ cells (cervical lymph nodes) were extracellularly stained with specific fluorochrome-conjugated monoclonal antibodies for 30 min at 4 °C. Macrophages were identified as CD45^+^MHCII^+^F4/80^+^ cells, and M1-like and M2-like subsets were gated as CD86^+^CD206^−/int^ or CD86^+^CD206^hi^ within the macrophage population, respectively (Table 1). CD8^+^ T cells were identified as CD45^+^, CD4^−^, and CD8α^+^ cells (Table 2). In addition, CD8^+^ T cells were stained intracellularly for IFN-γ and RANKL. For this, cells were stimulated with 50 ng/mL phorbol-12-myristate-13-acetate (PMA) and 1 µg/mL ionomycin (Merck) in the presence of 5 µg/mL brefeldin A (Biolegend) at 37 °C for 4 h. Cell fixation and permeabilization were performed using a Fixation/Permeabilization Staining kit (eBioscience, San Diego, CA, USA), and cells were stained overnight with specific fluorochrome-conjugated monoclonal antibodies (Table 2). Data were acquired using an LSRFortessa™ X-20 flow cytometer (Beckton Dickinson, Franklin Lakes, NJ, USA) and analyzed with FlowJo software v.10 (Beckton Dickinson). For macrophage identification, the gating strategy was established based on FSC-A/SSC-A parameters, FSC-H/FSC-A singlet discrimination, live/dead cell staining, and CD45, MHC class II, and F4/80 expression (Figure S1a). For CD8^+^ T cell identification, the gating strategy was established based on FSC-A/SSC-A parameters, FSC-H/FSC-A singlet discrimination, live/dead cell staining, and CD45, CD4, and CD8 expression (Figure S1b,c). All the experiments were performed separately.

### 4.7. RA-Treg EV Uptake and Macrophage Phenotype Assays

RA-Treg EV uptake and macrophage phenotypic characterization were analyzed simultaneously in vitro by flow cytometry. Briefly, RA-Treg EVs were labeled with 71 μM 1,1′-dioctadecyl-3,3,3′,3′-tetramethylindotricarbocyanine iodide (DiR; Biotium, CA, USA) for 1 h at 37 °C according to the manufacturer’s instructions. Following labeling, excess dye was removed by ultracentrifugation at 100,000× *g* for 1.5 h, and DiR-labeled RA-Treg EVs were resuspended in sterile PBS and stored at −20 °C until use within the same week. RAW264.7 macrophages were then seeded at a density of 2 × 10^5^ cells per well in 24-well plates and cultured overnight in complete DMEM supplemented with 10% FBS and antibiotics. The following day, macrophages were exposed to 1 × 10^8^ DiR-labeled RA-Treg EVs for 24 h. Macrophages stimulated with 100 ng/mL *E. coli* LPS were included as a pro-inflammatory positive control, whereas PBS-treated and DiR-PBS-treated macrophages were used as negative controls. Following stimulation, cells were harvested, washed extensively with PBS to remove unbound vesicles, and stained with fluorochrome-conjugated antibodies against CD86, MHC-II, CD68, iNOS, and Arg1 (Table 2). The uptake of RA-Treg EVs was assessed by DiR fluorescence, and macrophage phenotype was simultaneously evaluated by expression of polarization-associated markers. Data acquisition was performed using a Cytek Aurora spectral flow cytometer (Cytek Biosciences, Fremont, CA, USA), and data analysis was conducted using FlowJo software v.10 (Becton Dickinson, Franklin Lakes, NJ, USA). DiR fluorescence was used exclusively to assess EV association in DiR-RA-Treg EV- and DiR-PBS-treated macrophages, whereas phenotypic analyses were performed across all experimental groups, including PBS-, RA-Treg EV-, and LPS-treated cells. t-SNE analysis was performed to characterize macrophage phenotypic heterogeneity across experimental conditions.

### 4.8. CD8^+^ T-Cell Proliferation and Activation Assays

CD8^+^ T-cell proliferation and activation in the presence of RA-Treg EVs were analyzed in vitro by flow cytometry. Erythrocyte-depleted total leukocytes were obtained from the spleens of C57BL/6 wild-type mice. Briefly, spleens were harvested and mechanically disaggregated through a 70 µm nylon cell strainer using the plunger of a sterile syringe to generate single-cell suspensions. The cell suspensions were washed with PBS, centrifuged, and erythrocytes were removed using Red Blood Cell Lysis Buffer (BioLegend) according to the manufacturer’s instructions. After erythrocyte lysis, the remaining leukocytes were washed, resuspended in complete RPMI-1640 medium supplemented with 10% EV-depleted FBS, and counted using an automated cell counter. Leukocytes were then labeled with 5 µM CTV (Thermo Fisher Scientific) according to the manufacturer’s instructions. T cells were then polyclonally activated with 1 µg/mL soluble anti-CD3ε (Bio X Cell, Lebanon, NH, USA). Immediately after activation, 1 × 10^5^ responder T cells were cultured in the presence of 1 × 10^8^ RA-Treg EVs in RPMI-1640 medium supplemented with 10% EV-depleted FBS (Thermo Fisher Scientific). Non-activated T cells or activated but untreated T cells (exposed to the vehicle PBS without RA-Treg EVs) were used as controls. After 72 h, T cells were stained for CD4, CD8, and CD25 using fluoro-chrome-conjugated monoclonal antibodies (Table 3). The effect of RA-Treg EVs on CD8^+^ T-cell proliferation and activation was evaluated by assessing the CTV dilution profile and CD25 expression, respectively. Data acquisition was performed using a FACS Canto II flow cytometer (BD Biosciences), and analyses were conducted with FlowJo software v.10 (BD Biosciences).

### 4.9. Data Analysis

The sample size was determined using G*Power software v.3.1 (Heinrich-Heine-Universität Düsseldorf, Düsseldorf, Germany), with a significance level of 5% (α = 0.05) and a statistical power of 80% (1 − β = 0.80). Alveolar bone loss was considered the primary outcome variable. The sample size was calculated to detect a minimum difference of 185.96 µm^2^ in alveolar bone loss between untreated periodontitis mice and RA-Treg EV-treated periodontitis mice, assuming a standard deviation of 113.4 µm^2^ [37]. This calculation yielded a minimum sample size of 6 animals per experimental group, for a total of 18 animals.

Quantitative data derived from micro-CT and flow cytometry were expressed as mean ± standard deviation or as median with interquartile range boxes, and whiskers indicating the 10th and 90th percentiles. All statistical analyses were performed using R software version 2026.01.0 (Posit Software PBC, Boston, MA, USA). Analyses were conducted in a blinded manner, and each animal was considered an independent experimental unit. The normality of data distribution was assessed using the Shapiro–Wilk test. The homocedasticity of the data was assessed using the Levene test. Statistical differences were determined using one-way ANOVA followed by Bonferroni’s multiple comparisons test, one-way ANOVA with Welch’s correction followed by Bonferroni’s multiple comparisons test, or Kruskal–Wallis test followed by Wilcoxon post hoc comparisons with Bonferroni adjustment, depending on the data distribution and homocedasticity. The level of significance was set at α < 0.05.

## 5. Conclusions

Local administration of RA-Treg EVs attenuated ligature-induced alveolar bone loss and preserved trabecular microarchitecture in experimental periodontitis. This bone-protective effect was associated with reduced macrophage infiltration into periodontal tissues, restoration of CD8^+^ T-lymphocyte abundance in periodontal tissues and draining cervical lymph nodes, and direct enhancement of CD8^+^ T-cell proliferation and activation in vitro. Collectively, these findings indicate that RA-Treg EVs attenuate alveolar bone loss during experimental periodontitis and are associated with selective modulation of immune cell populations involved in periodontal inflammation, including macrophages and CD8^+^ T lymphocytes. However, whether the restoration of CD8^+^ T-cell abundance directly contributes to the bone-protective effects of RA-Treg EVs remains to be determined in future mechanistic studies.

## Figures and Tables

**Table 1:** Monoclonal antibodies and cell viability kit used for in vivo macrophage detection.

Antibody	Clone	Fluorochrome	Code	Supplier
anti-CD45	30-F11	BUV737 ^2^	748371	BD Biosciences ^8^
anti-MHCII	M5/114-15.2	PE ^3^	107607	Biolegend ^9^
anti-F4/80	BM8	APC ^4^	123115	Biolegend
anti-CD86	GL-1	FITC ^5^	105005	Biolegend
anti-CD206	C068C2	PE Cy7 ^6^	141719	Biolegend
Viability ^1^	--	NIR ^7^	423105	Biolegend

^1^ Cell viability kit: Zombie NIR Fixable Viability Kit; ^2^ BUV: Brilliant Ultra Violet™; ^3^ PE: Phycoerythrin; ^4^ APC: Allophycocyanin; ^5^ FITC: Fluorescein isothiocyanate; ^6^ PE-Cy7: Phycoerythrin-Cyanine7; ^7^ NIR: Near Infrared; ^8^ BD Biosciences, San Jose, CA, USA. ^9^ Biolegend, San Diego, CA, USA.

**Table 2:** Monoclonal antibodies and cell viability kit used for CD8^+^ T lymphocyte detection.

Antibody	Clone	Fluorochrome	Code	Supplier
anti-CD4	GK1.5	BV605 ^2^	100451	Biolegend ^6^
anti-CD8α	53-6.7	BUV396 ^3^	563786	BD Biosciences ^7^
anti-CD25	PC61	BV421	102034	Biolegend
anti-CD45	30-F11	BV711	103147	Biolegend
anti-IFN-γ	XMG1.2	APC ^4^	505810	Biolegend
anti-Foxp3	150D	Alexa Fluor 488	320012	Biolegend
anti-RANKL	IK22/5	PE ^5^	510006	Biolegend
Viability ^1^	--	BUV496	423108	Biolegend

^1^ Cell viability kit: Zombie UV Fixable Viability Kit; ^2^ BV: Brilliant Violet™; ^3^ BUV: Brilliant Ultra Violet™; ^4^ APC: Allophycocyanin; ^5^ PE: Phycoerythrin; ^6^ Biolegend, San Diego, CA, USA; ^7^ BD Biosciences, San Jose, CA, USA.

**Table 3:** Monoclonal antibodies and cell viability kit used for in vitro macrophages characterization.

Antibody	Clone	Fluorochrome	Code	Supplier
anti-CD45	30-F11	BV711 ^2^	103147	Biolegend
anti-CD11b	M1/70	Pacific Blue	101224	Biolegend ^6^
anti-MHCII	M5/114.15.2	PE Dazzle^TM^ 594	107647	Biolegend
anti-CD86	GL-1	FITC	105006	Biolegend
anti-CD68	FA-11	PerCP Cy5.5 ^3^	137009	Biolegend
anti-iNOS	W16030C	PE Cy7 ^4^	696814	Biolegend
anti-Arg1	W21047I	PE ^5^	165804	Biolegend
Viability ^1^	--	BV510	13-0870-T100	Cytek Biosciences ^7^

^1^ Cell viability kit: Ghost Dye^®^ Violet 510; ^2^ BV: Brilliant Violet™; ^3^ PerCP-Cy5.5: Peridinin-chlorophyll-protein complex-Cyanine5.5; ^4^ PE-Cy7: Phycoerythrin-Cyanine7; ^5^ PE: Phycoerythrin; ^6^ Biolegend, San Diego, CA, USA; ^7^ Cytek Biosciences, Fremont, CA, USA.

## Data Availability

The original contributions presented in the study are included in the article. Further inquiries can be directed to the corresponding authors.

## Supplementary Materials

The following supporting information can be downloaded at: https://www.mdpi.com/article/10.3390/ijms27135845/s1.

## Abbreviations

The following abbreviations are used in this manuscript:

APCs	Antigen-presenting cells
Arg1	Arginase-1
CD	Cluster of differentiation
CEJ	Cement-enamel junction
CTV	Cell trace violet
DiR	1,1′-dioctadecyl-3,3,3′,3′-tetramethylindotricarbocyanine iodide
EDTA	Ethylenediaminetetraacetic acid
EVs	Extracellular vesicles
FBS	Fetal bovine serum
IFN-γ	Interferon-γ
IL-2	Interleukin-2
iNOS	Inducible nitric oxide synthase
LPS	Lipopolysaccharide
Micro-CT	Micro-computed tomography
MHC	Major histocompatibility complex
NTA	Nanoparticle tracking analysis
PBS	Phosphate-buffered saline
PMA	Phorbol-12-myristate-13-acetate
RA	Retinoic acid
RANKL	Receptor activator fn nuclear factor κB ligand
ROI	Region of interest
TGF-β1	Transforming growth factor-β1
Tregs	T regulatory lymphocytes
t-SNE	t-distributed stochastic neighbor embedding
VOI	Volume of interest

## References

[1] Alvarez C., Monasterio G., Cavalla F., Cordova L.A., Hernandez M., Heymann D., Garlet G.P., Sorsa T., Parnanen P., Lee H.M. (2019). Osteoimmunology of oral and maxillofacial diseases: Translational applications based on biological mechanisms. Front. Immunol..

[2] Hajishengallis G., Lamont R.J. (2021). Polymicrobial communities in periodontal disease: Their quasi-organismal nature and dialogue with the host. Periodontol 2000.

[3] Rojas C., García M., González-Osuna L., Campos-Mora M., de León E.P., Sierra-Cristancho A., Terraza C., Cortez C., Sansores-España L.D., Carvajal P. (2025). Induced Treg-derived extracellular vesicles suppress CD4^+^ T-cell-mediated inflammation and ameliorate bone loss during periodontitis partly through CD73/Adenosine-dependent immunomodulatory mechanisms. J. Extracell. Vesicles.

[4] Hajishengallis G., Lamont R.J. (2016). Dancing with the Stars: How Choreographed Bacterial Interactions Dictate Nososymbiocity and Give Rise to Keystone Pathogens, Accessory Pathogens, and Pathobionts. Trends Microbiol..

[5] Zhang M., Liu Y., Afzali H., Graves D.T. (2024). An update on periodontal inflammation and bone loss. Front. Immunol..

[6] Ginesin O., Mayer Y., Gabay E., Rotenberg D., Machtei E.E., Coyac B.R., Bar-On Y., Zigdon-Giladi H. (2023). Revealing leukocyte populations in human peri-implantitis and periodontitis using flow cytometry. Clin. Oral Investig..

[7] Qiu Y., Ding Z., Yang D. (2025). A bibliometric analysis of macrophage research associated with periodontitis over the past two decades. Int. Dent. J..

[8] Han Y.K., Jin Y., Miao Y.B., Shi T., Lin X.P. (2018). CD8^+^ Foxp3^+^ T cells affect alveolar bone homeostasis via modulating Tregs/Th17 during induced periodontitis: An adoptive transfer experiment. Inflammation.

[9] Kayar N.A., Çelik I., Gözlü M., Üstün K., Gürsel M., Alptekin N.O. (2024). Immunologic burden links periodontitis to acute coronary syndrome: Levels of CD4^+^ and CD8^+^ T cells in gingival granulation tissue. Clin. Oral Investig..

[10] Baima G., Arce M., Romandini M., Van Dyke T. (2025). Inflammatory and immunological basis of periodontal diseases. J. Periodontal Res..

[11] Yamaguchi T. (2026). Functions of macrophages in periodontal disease progression and gingival tissue homeostasis. Adv. Exp. Med. Biol..

[12] Al Moussawy M., Abdelsamed H.A. (2022). Non-cytotoxic functions of CD8 T cells: “Repentance of a serial killer”. Front. Immunol..

[13] Shashkova E.V., Trivedi J., Cline-Smith A.B., Ferris C., Buchwald Z.S., Gibbs J., Novack D., Aurora R. (2016). Osteoclast-primed Foxp3^+^ CD8 T cells induce T-bet, eomesodermin, and IFN-γ to regulate bone resorption. J. Immunol..

[14] Buchwald Z.S., Yang C., Nellore S., Shashkova E.V., Davis J.L., Cline A., Ko J., Novack D.V., DiPaolo R., Aurora R. (2015). A bone anabolic effect of RANKL in a murine model of osteoporosis mediated through FoxP3^+^ CD8 T cells. J. Bone Miner. Res..

[15] Terauchi M., Li J.Y., Bedi B., Baek K.H., Tawfeek H., Galley S., Gilbert L., Nanes M.S., Zayzafoon M., Guldberg R. (2009). T lymphocytes amplify the anabolic activity of parathyroid hormone through Wnt10b signaling. Cell Metab..

[16] Tyagi A.M., Yu M., Darby T.M., Vaccaro C., Li J.Y., Owens J.A., Hsu E., Adams J., Weitzmann M.N., Jones R.M. (2018). The microbial metabolite butyrate stimulates bone formation via T regulatory cell-mediated regulation of Wnt10b expression. Immunity.

[17] Wolf M., Lossdörfer S., Marciniak J., Römer P., Kirschneck C., Craveiro R., Deschner J., Jäger A. (2016). CD8^+^ T cells mediate the regenerative PTH effect in hPDL cells via Wnt10b signaling. Innate Immun..

[18] Roser-Page S., Weiss D., Vikulina T., Yu M., Pacifici R., Weitzmann M.N. (2022). Cyclic adenosine monophosphate (cAMP)-dependent phosphodiesterase inhibition promotes bone anabolism through CD8^+^ T cell Wnt-10b production in mice. JBMR Plus.

[19] Yang D., He D., Yang F., Meng X., Zheng K., Lin H., Cheng Y., Tam W.C., Li G. (2025). Advances in harnessing biological macromolecules for periodontal tissue regeneration: A review. Int. J. Biol. Macromol..

[20] Liu G., Xue J., Zhou X., Gui M., Xia R., Zhang Y., Cai Y., Li S., Shi S., Mao X. (2025). The paradigm shifts of periodontal regeneration strategy: From reparative manipulation to developmental engineering. Bioact. Mater..

[21] Cafferata E.A., Jerez A., Vernal R., Monasterio G., Pandis N., Faggion C.M. (2019). The therapeutic potential of regulatory T lymphocytes in periodontitis: A systematic review. J. Periodontal Res..

[22] Zhang Y., Guo J., Jia R. (2021). Treg: A promising immunotherapeutic target in oral diseases. Front. Immunol..

[23] Rojas C., Campos-Mora M., Cárcamo I., Villalón N., Elhusseiny A., Contreras-Kallens P., Refisch A., Gálvez-Jirón F., Emparán I., Montoya-Riveros A. (2020). T regulatory cells-derived extracellular vesicles and their contribution to the generation of immune tolerance. J. Leukoc. Biol..

[24] Cavalla F., Hernández M. (2022). Polarization profiles of T lymphocytes and macrophages responses in periodontitis. Adv. Exp. Med. Biol..

[25] Xu H., Wang T., Yang Y. (2025). BPIFA1 inhibits periodontitis by regulating the NF-κB/IκB signaling pathway and macrophage M1/M2 polarization. Arch. Oral Biol..

[26] Miao Y., He L., Qi X., Lin X. (2020). Injecting immunosuppressive M2 macrophages alleviates the symptoms of periodontitis in mice. Front. Mol. Biosci..

[27] Popovic P.J., Zeh H.J., Ochoa J.B. (2007). Arginine and immunity. J. Nutr..

[28] Song L., Lee C., Schindler C. (2011). Deletion of the murine scavenger receptor CD68. J. Lipid Res..

[29] Rabinowitz S.S., Gordon S. (1991). Macrosialin, a macrophage-restricted membrane sialoprotein differentially glycosylated in response to inflammatory stimuli. J. Exp. Med..

[30] Lipuma J.J. (2010). The changing microbial epidemiology in cystic fibrosis. Clin. Microbiol. Rev..

[31] Gaur T., Lengner C.J., Hovhannisyan H., Bhat R.A., Bodine P.V., Komm B.S., Javed A., van Wijnen A.J., Stein J.L., Stein G.S. (2005). Canonical WNT signaling promotes osteogenesis by directly stimulating Runx2 gene expression. J. Biol. Chem..

[32] Asemani Y., Najafi S., Ezzatifar F., Zolbanin N.M., Jafari R. (2022). Recent highlights in the immunomodulatory aspects of Treg cell-derived extracellular vesicles: Special emphasis on autoimmune diseases and transplantation. Cell Biosci..

[33] Moya-Guzmán M.J., González-Mienert E., Pinto C., Pino-Lagos K. (2026). TGF-β-induced Tregs release extracellular vesicles that modulate CD4^+^ T-cell proliferation and phenotype in a Nrp1-dependent manner. Immunohorizons.

[34] Lu J., Wu J., Tian J., Wang S. (2018). Role of T cell-derived exosomes in immunoregulation. Immunol. Res..

[35] Zhang X., Gao H., Lin L. (2025). The extracellular vesicle-based treatment: A developing strategy for periodontal diseases. Front. Immunol..

[36] Xia E.J., Zou S., Zhao X., Liu W., Zhang Y., Zhao I.S. (2024). Extracellular vesicles as therapeutic tools in regenerative dentistry. Stem Cell Res. Ther..

[37] Cafferata E.A., Castro-Saavedra S., Fuentes-Barros G., Melgar-Rodriguez S., Rivera F., Carvajal P., Hernandez M., Cortes B.I., Cortez C., Cassels B.K. (2021). Boldine inhibits the alveolar bone resorption during ligature-induced periodontitis by modulating the Th17/Treg imbalance. J. Periodontol..

[38] Monasterio G., Budini V., Fernandez B., Castillo F., Rojas C., Alvarez C., Cafferata E.A., Vicencio E., Cortes B.I., Cortez C. (2019). IL-22-expressing CD4^+^ AhR^+^ T lymphocytes are associated with RANKL-mediated alveolar bone resorption during experimental periodontitis. J. Periodontal Res..

[39] Monasterio G., Castillo F., Ibarra J.P., Guevara J., Rojas L., Alvarez C., Fernandez B., Aguero A., Betancur D., Vernal R. (2018). Alveolar bone resorption and Th1/Th17-associated immune response triggered during Aggregatibacter actinomycetemcomitans-induced experimental periodontitis are serotype-dependent. J. Periodontol..

