# Supplementary material for: Targeting Periodontitis with Treg-Derived Extracellular Vesicles: Modulation of Macrophages and CD8+ T-Cell Responses
Source: Int J Mol Sci. 2026 Jun 29;27(13):5845. doi: 10.3390/ijms27135845 (PMC13362477; doi:10.3390/ijms27135845)
Supplement: Supplementary file 1 [file ijms-27-05845-s001.zip › ijms-4296529-supplementary.pdf]

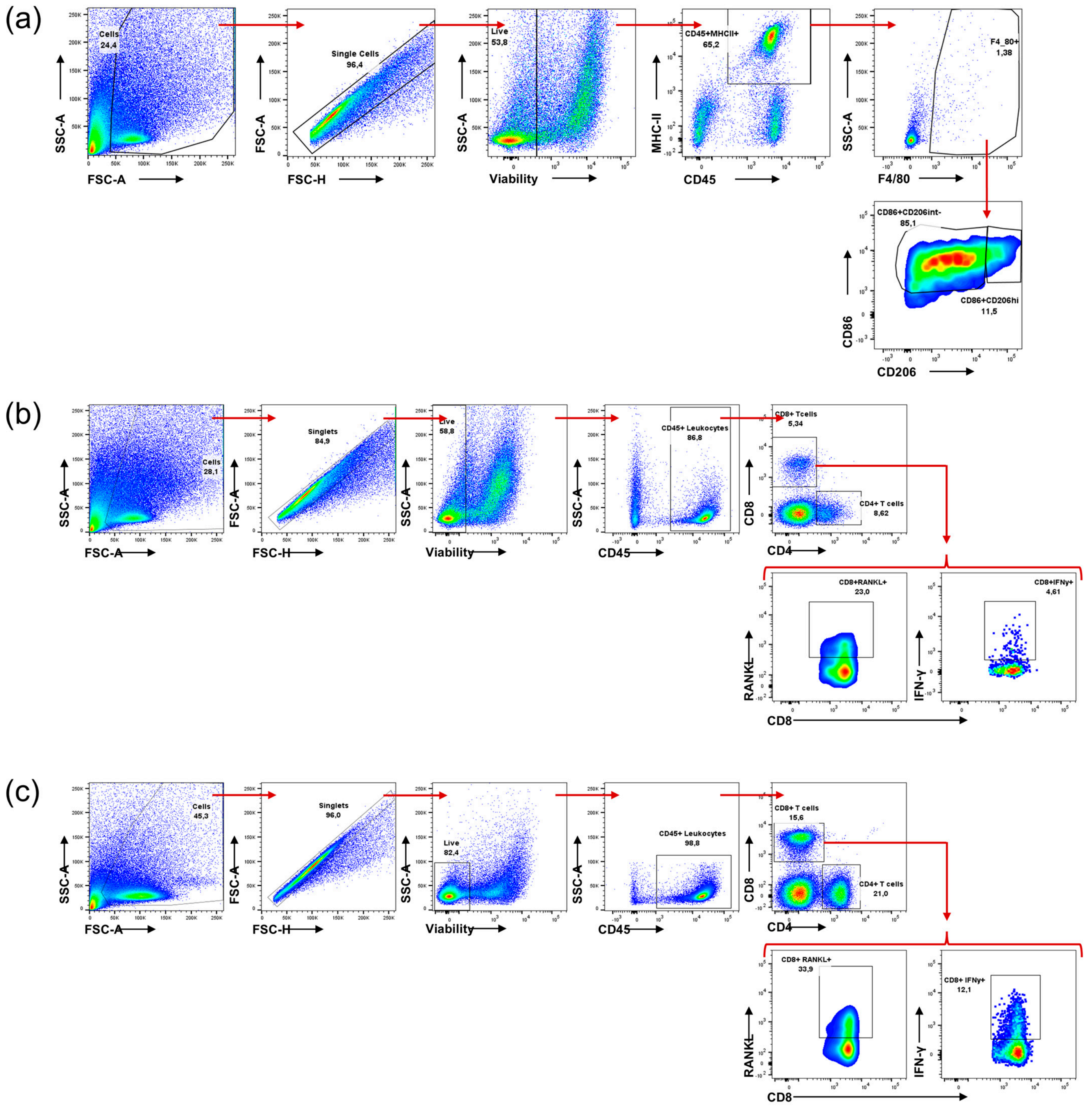

Figure S1: Gating strategies used to identify and quantify periodontal-infiltrating macrophages and CD8+ T lymphocytes by flow cytometry.

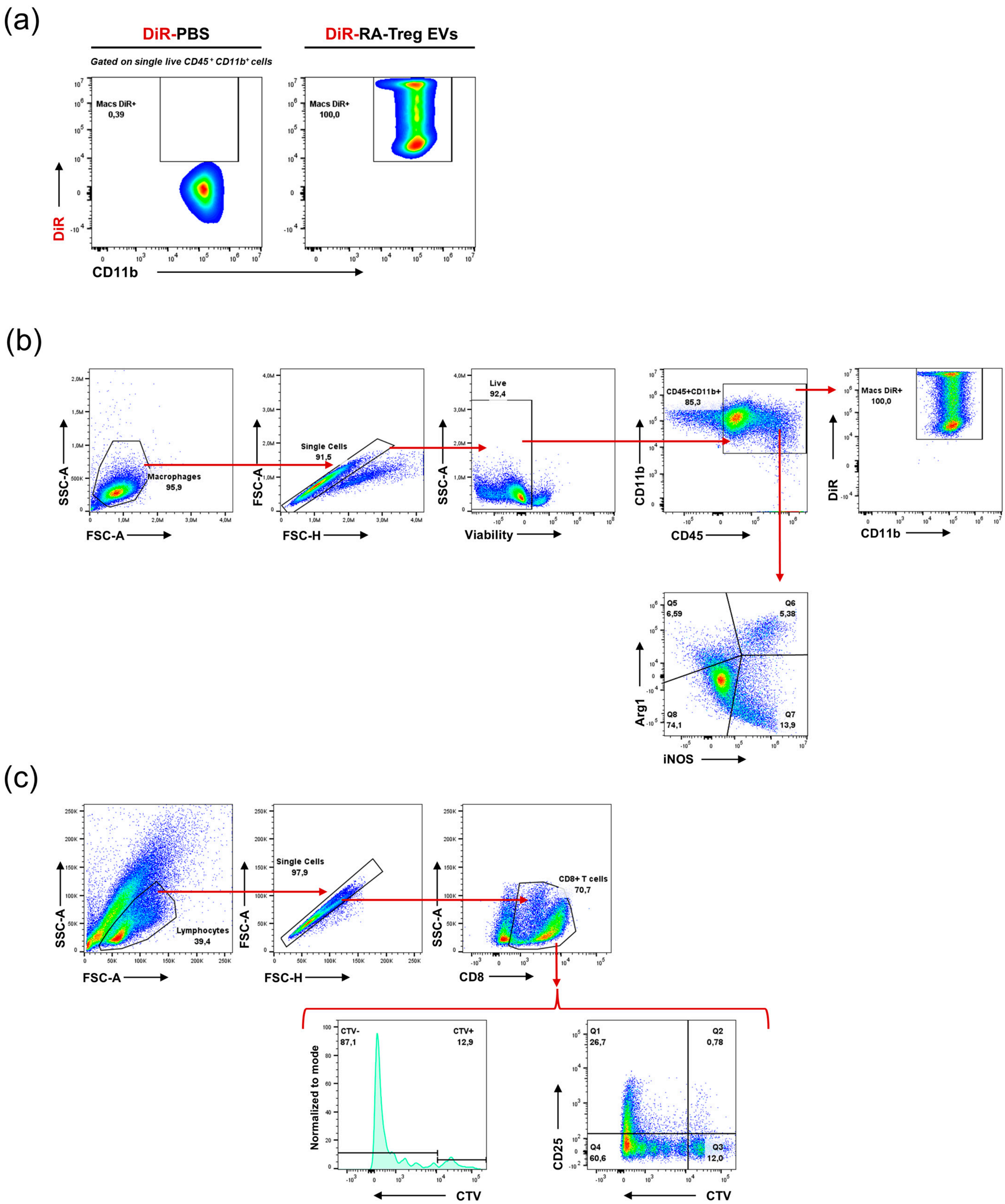

Figure S2a: Representative flow cytometry dot plots showing the uptake of DiR-labeled RA-Treg EVs by RAW264.7 macrophages after exposure to DiR-PBS (control) or DiR-labeled RA-Treg EVs. Figure S2b,c: Gating strategy used to identify and quantify macrophages and CD8+ T lymphocytes *in vitro* by flow cytometry.
